# Functional hydrogels for diabetic wound management

**DOI:** 10.1063/5.0046682

**Published:** 2021-07-09

**Authors:** Daqian Gao, Yidan Zhang, Daniel T. Bowers, Wanjun Liu, Minglin Ma

**Affiliations:** Department of Biological and Environmental Engineering, Cornell University, Ithaca, New York 14853, USA

## Abstract

Diabetic wounds often have a slow healing process and become easily infected owing to hyperglycemia in wound beds. Once planktonic bacterial cells develop into biofilms, the diabetic wound becomes more resistant to treatment. Although it remains challenging to accelerate healing in a diabetic wound due to complex pathology, including bacterial infection, high reactive oxygen species, chronic inflammation, and impaired angiogenesis, the development of multifunctional hydrogels is a promising strategy. Multiple functions, including antibacterial, pro-angiogenesis, and overall pro-healing, are high priorities. Here, design strategies, mechanisms of action, performance, and application of functional hydrogels are systematically discussed. The unique properties of hydrogels, including bactericidal and wound healing promotive effects, are reviewed. Considering the clinical need, stimuli-responsive and multifunctional hydrogels that can accelerate diabetic wound healing are likely to form an important part of future diabetic wound management.

## I. INTRODUCTION

Skin, the largest organ of the human body, comprises epidermis, dermis, and hypodermis.^1^ It is the first barrier to external invasion.^2^ Injured skin can be categorized into normal or chronic wounds.^3^ A healthy wound healing process involves four overlapping but independent phases.^3–6^ First, growth factors and cytokines are released during the hemostasis phase, immediately after injury.^7^ Second, an inflammation phase follows hemostasis within hours, allowing immune cells, such as neutrophils, to infiltrate the injury site to kill bacteria and remove damaged matrix proteins.^6^ Third, the proliferation phase comes after the inflammation phase. Various growth factors, including platelet-derived growth factor (PDGF), vascular endothelial growth factor (VEGF), and fibroblast growth factors (FGFs) released by newly produced cells stimulate epidermal repair and angiogenesis to form soft and pink granulation tissue.^3^ Finally, an extracellular matrix (ECM) remodeling phase involves replacing type III collagen of the dermis with type I collagen, resulting in scar tissue formation with rich collagen fibers.^8^ In contrast to a normal wound healing that resolves within two–three months,^9^ the healing period of chronic wounds can exceed three months because most of these wounds fail to progress to the proliferation phase and suffer from chronic inflammation.^10,11^

In recent years, 9.5% of the world population was reported to be affected by diabetes, and the incidence is still increasing.^12^ A diabetic patient has a 15%–20% lifetime risk to develop a diabetic foot ulcer.^13^ Diabetic ulcers are a common type of chronic wound Ulcers impact patients' quality of life and require costly treatments.^4,14^ The diabetic ulcer has an exceptionally complex pathology due to persistent hyperglycemia and associated diabetic complications. The difficult-to-heal fact of diabetic wounds can be summarized as follows. (1) Many diabetic wounds are infected. Hyperglycemia promotes bacterial colonization and biofilm formation, leading to uncontrollable inflammation.^15^ Moreover, biofilms are found in most ulcers, exacerbating the already unstable wound environments.^14,16^ (2) High oxidative stress, mainly induced by excessive reactive oxygen species (ROS), often causes sustained inflammation.^15,17^ ROS are generated by cells, such as inflammatory cells, epithelial cells, and endothelial cells after injuries.^18^ Persistent hyperglycemia in diabetic wounds produces advanced glycation end products (AGEs) in the blood. AGEs then directly induce overproduction of ROS.^17,19,20^ In addition to leading to chronic inflammation, excessive ROS also impairs angiogenesis, promotes senescent cells, and hinders re-epithelization.^21^ (3) Microvascular complications of diabetes cause vascular damage and ischemia, leading to deficiencies in oxygen and nutrient delivery to wound sites.^3,22^ As a primary wound care practice, wound dressings are commonly used to cover, protect, and facilitate the healing of wounds. Materials designed specifically as wound dressings can assist throughout the wound healing process. Maintaining moist conditions in wound sites is crucial and challenging in wound management.^23^ Since the work of Hinman and Marbach, the modern wound healing concept has switched from a dry wound bed to a moist wound healing model.^24^ It is now commonly accepted that an ideal condition for tissue regeneration should have a balanced moisture,^25^ i.e., neither flooded with wound exudates nor dried out due to lack of moist supply. The inherent water content of hydrogels is an advantage compared to other wound dressing materials. Being a 3D matrix, hydrogels can easily load drugs or cytokines specifically aimed at major difficulties in diabetic wounds. To increase the chance of wound healing, antimicrobial agents^26–28^ and glucose control drugs^29–33^ are often loaded. ROS-scavenging compounds^28,34^ and angiogenesis promoting compounds^28,35^ are also loaded in hydrogels to resolve the above-mentioned obstacles throughout diabetic wound healing.

Most diabetic wounds have bacterial infections because the hyperglycemia of the wound bed provides an ideal environment for the growth of microorganisms.^3,36^ The bacteria can then develop into a biofilm after attachment, which drives wounds into a long-lasting inflammatory phase.^37^ Once biofilms form, it is a difficult challenge to clear infections due to the robust resistance toward treatments, especially small molecule antibiotics. It was reported that more than 78% of chronic wounds had biofilms.^38^ In a small-scale clinical study of chronic wounds, out of 50 chronic wound specimens from patients, 30 were found to contain biofilms.^39^ The persistently infected chronic wounds in diabetic patients can often cause serious outcomes: 14%–24% of patients with diabetic ulcers are estimated to eventually require amputation.^40,41^ Thus, combating infections and biofilms is crucial to improve the diabetic patients' quality of life.

Wound debridement, weight control, and patient education are part of the current clinical treatment for diabetic ulcers.^42^ However, these treatments do not address the issues about bacterial infection, excessive ROS, and impaired angiogenesis, nor do they accelerate wound healing.^43^ To solve these problems, new therapeutic approaches, including wound autografts, wound dressings, and tissue engineering scaffolds, have been extensively explored. However, Food and Drug Administration (FDA) approved biomaterials for the treatment of diabetic wounds are limited. Nevertheless, numerous biomaterials have supported positive research outcomes, such as poly (lactic-co-glycolic acid) (PLGA), collagen, chitosan (CS), and others. The polymers can be processed into nano- or microparticles, electrospun nanofibers, bandages, sponges, and hydrogels as drug delivery systems or wound dressings for diabetic wound healing. Among them, hydrogels have been studied intensively due to their biocompatibility and possibility of functionalization. Hydrogels are 3D porous material networks with high water content. Drugs or drug-like components, such as antibiotics, inorganic nanoparticles, antioxidants, growth factors, and cells, can be encapsulated into hydrogel matrices, often to achieve sustained release. Hydrogels are thus considered as ideal candidates for diabetic wound dressings.^44–46^ For example, compared to particles and electrospun nanofiber films, hydrogels provide 3D architectures for cell migration and tissue regeneration. Their water retention ability provides a wet environment for wound healing, which sponges or conventional bandages do not have.

**FIG. 1.**
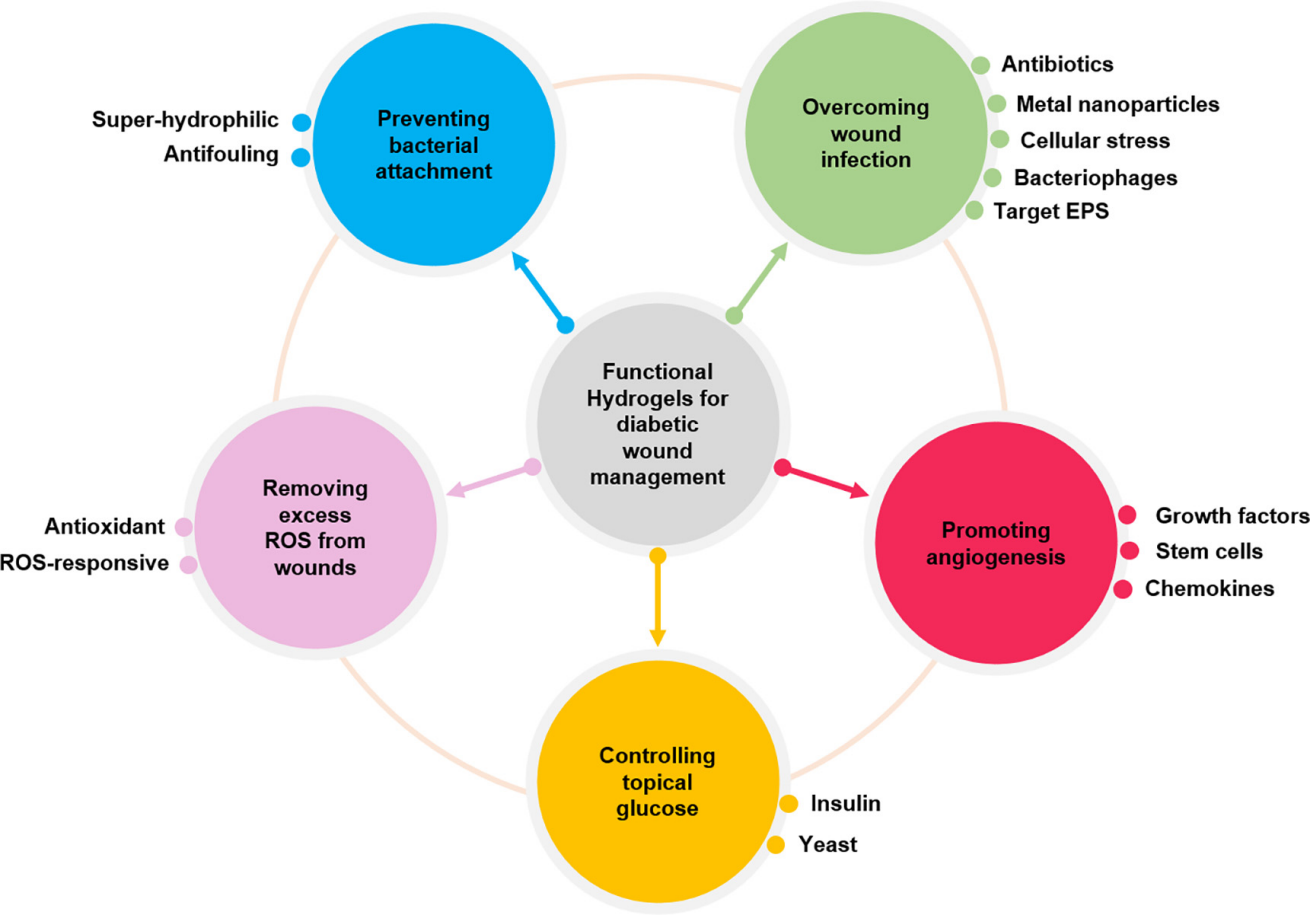
Functional hydrogels for diabetic wound management.

In this review, recent research progress in managing diabetic wounds will be reviewed with a special focus on how researchers have designed novel hydrogel dressings to solve biofilm-related infections and promote diabetic wound healing. The main goal of this review is to introduce the role of hydrogels in the healing process of diabetic ulcers from the perspective of biomaterial design. It highlights hydrogel dressings in different aspects during diabetic wound healing, including mitigation of infections and excessive ROS as well as promotion of angiogenesis. In addition, multifunctional hydrogels with antibacterial function and the ability to sense or control topical pH or glucose levels can be fabricated to promote diabetic wound healing (Fig. 1).

## II. HYDROGELS AGAINST INFECTIONS

Biofilms are made of an aggregated population of bacteria within a 3D matrix of self-secreted biopolymers.^47^ Biofilms typically form in four steps: attachment, reproduction, development, and colonization. The attachment happens when planktonic bacteria (that is, free-floating bacteria) start to touch a surface. After attachment, bacteria can reproduce quickly due to the high glucose environment and inhibited local immune response caused by the disorder of pro- and anti-inflammatory cytokines.^48^ Meanwhile, bacteria also start to develop biofilms by secreting extracellular polymeric substances (EPS), including polysaccharides, proteins, nucleic acids, lipids, etc.^49^ The EPSs can be as much as 90% of the total weight in a biofilm and play an essential role in biofilm resistance toward countermeasures.^47^ Once developed, a biofilm becomes hard to destroy. The last step is to colonize. A biofilm is a dynamic system that can release planktonic bacteria into surrounding environments.^50^ Once the released bacteria find their suitable sites, new biofilms will form following the same processes. It is noteworthy that the biofilms may be proceeding at different stages for multiple bacterial species, and other bacteria can also attach to the surface and colonize. Competition and cooperation in a diverse population often give rise to resistance to environmental changes and treatments.

In addition to bacterial population diversity, the unique EPS matrix of biofilms contributes to its resistance. The spatial proximity of bacteria cells living in an EPS matrix ensures good cell–cell communication, leading to an adaptable system.^51^ The EPS matrix can work as a buffering and organizing center that on one hand facilitates nutrient supply and on the other hand handles waste recycling.^52^ The difficult-to-degrade nature of the EPS matrix offers sufficient protection for bacteria against host-immune responses, by preventing physical contact, and hinders the penetration of treatments.^53^

To overcome the wound infections of diabetic ulcers, functional hydrogels have been designed and investigated. One strategy is using antibacterial hydrogels to kill bacteria. Hydrogels with inherent antibacterial properties or hydrogels loaded with antibiotics or drug-like components can be used to reduce bacterial viability. When biofilms exist, hydrogels designed to remove EPSs may also be needed. Another strategy is using antifouling hydrogels to prevent planktonic cells from initial attachment, thereby reducing the formation of biofilms.

### A. Hydrogels against bacterial attachment

Accumulation of dead bacteria may compromise antibacterial functions, especially for those based on contact killing mechanisms. Therefore, hydrogels capable of preventing initial bacterial attachment are highly desired. The initial nonspecific binding of bacteria is a dynamic equilibrium that involves constant adsorption and detachment of proteins. In general, both superhydrophobic and superhydrophilic surfaces have been investigated to prevent bacterial attachment. Although not yet fully demonstrated, the well-observed antifouling effects of superhydrophobic surfaces are believed to be attributed to the large surface contact angle. To be more specific, liquid droplets easily roll off superhydrophobic surfaces, which potentially promotes the detachment of bacteria and reduces nonspecific surface binding.^54^ Other hypotheses about the antifouling mechanism of superhydrophobic surfaces include less available binding sites due to air trapped in the interfaces, conformational changes in adsorbed proteins, and selective protein adsorption/denaturation.^55,56^ However, the superhydrophobic design is rarely used in the form of hydrogels. This is mostly because the hydrophilic nature of hydrogels makes it difficult, although not impossible, to achieve a stable superhydrophobic surface on hydrogels.

**FIG. 2.**
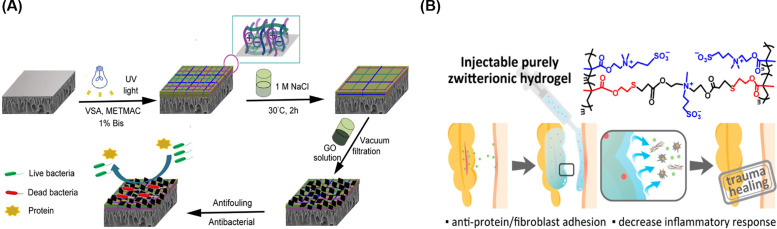
Zwitterionic hydrogels for preventing bacterial attachment: (a) a zwitterionic polyampholyte hydrogel was grafted on a polyethersulfone (PES) surface to provide antifouling properties.^69^ Reprinted with permission from Wang *et al.*, J. Membr. Sci. **565**, 293–302 (2018). Copyright 2018 Elsevier. (b) A zwitterionic hydrogel crosslinked via the thiol–ene click reaction resisted adhesion of protein and cells.^73^ Reproduced with permission from Guo *et al.*, Chem. Mater. **32**(15), 6347–6357 (2020). Copyright 2020 American Chemical Society.

Unlike hydrophobic surfaces, superhydrophilic surfaces can inhibit biofouling because physical and energetic barriers form after water molecules bind to such surfaces. Such hydration barriers can prevent the initial nonspecific protein adsorption onto superhydrophilic surfaces, which has been proven to prevent the initial step of planktonic bacterial attachment.^57^ Apart from well-known hydrophilic polymers, such as hyaluronic acid (HA), poly (ethylene glycol) (PEG), and peptides, zwitterionic polymers, such as polycarboxybetaine (PCB),^58–60^ polysulfobetaine (PSB),^61–63^ poly (quaternized triazole carboxybetaine acrylamide) [P(qTR-CB)],^64^ and poly(2-methacryloyloxyethyl phosphorylcholine) (PMPC), appear to have superior antifouling properties due to their excellent water-binding capability and thus superhydrophilicity.^65,66^ Traditional hydrophilic surfaces, represented by PEG and its derivatives, are achieved by hydrogen bonds between surfaces and water molecules. In contrast, zwitterionic polymers can maintain a more stable hydration layer on the surface by electrostatic interactions between zwitterions and water molecules,^63,67^ leading to superior antifouling properties to prevent the attachment of bacteria.^61,62,68^ For example, the Bernstein lab demonstrated the antifouling properties of their zwitterionic polyampholyte grafted poly (ether sulfone) (PES) surfaces [Fig. 2(a)].^69^ The zwitterionic polyampholyte was synthesized by vinylsulfonic acid (VSA) with negative charges and [2-(methacryloyloxy) ethyl] trimethylammonium chloride solution (METMAC) with positive charges.^70^ After modification, the contact angle of the PES surfaces showed a decrease from 78° ± 4° to 63° ± 3°. In addition, 70% decrease in protein adsorption and 80% decrease in *E. Coli* colonies were observed when PES surface was coated with the zwitterionic hydrogels. Inspiringly, synergistic effects have been found when combining traditional and zwitterionic antifouling strategies. For example, Xia *et al.* coated a gold surface with hyaluronic acid (HA), zwitterionic “bottle-brush polymer” (BB), and the combination of HA and BB.^71^ The bovine serum albumin (BSA) adsorption was lowered to 0.2 ± 0.3 ng/cm^2^ when combined, less than 1/60th of those modified with HA or BB alone.

Besides preventing bacterial attachment, antifouling hydrogels can also prevent the adhesion between newly formed granulation tissue and hydrogel dressings when the dressings are changed. Liu *et al.* demonstrated complete elimination of *in vitro* endothelial cell attachment when the surface was modified with a serine-based zwitterionic polymer.^72^ When tested *in vivo*, Guo *et al.* successfully created a zwitterionic hydrogel that was clickable via a thiol–ene click reaction, which showed level 0 (no adhesion) in their adhesion scoring system, a significant improvement compared to level 2 (more than one thin adhesion) for hyaluronic hydrogel [Fig. 2(b)].^73^ The antiadhesive feature of such hydrogels, although having limited contribution to wound healing, largely reduces patients' pain upon change of dressings. Additionally, the antiadhesive hydrogels reduce secondary tissue damage so that the healing process would not be disrupted.

Overall, antifouling hydrogels are mostly preventative dressings. Antiadhesion makes the chronic wound healing process less disrupted and improves patients' experience during the change of dressings. Although the latest techniques can achieve satisfying effects against bacterial adhesion, once wounds are infected, hydrogels must incorporate antibacterial properties to treat infections. In Sec. II B, we will discuss hydrogels with antimicrobial properties and the design of such dressings to eliminate bacteria when the wound sites are already infected.

### B. Hydrogels with antibacterial properties

Chitosan^74^ and some antimicrobial peptides^75^ with inherent antibacterial effects can be made into hydrogels. However, these hydrogels rely on contact killing requiring close contact for hours, which is rarely sufficient to eradicate infections.^76^ Therefore, loading hydrogels with antibacterial components has been widely accepted to enhance hydrogels' antibacterial properties. By using hydrogels as a carrier, the controllable release of antibacterial components can be achieved. In addition, antibacterial components could be locally delivered to the entire wound area, rendering a much better antibacterial efficacy. Furthermore, various antibacterial compounds are available, such as antibiotics, metal and metallic oxide nanoparticles, and phages. Each of these antibacterial compounds has its advantages over others. Hydrogels offer a delivery platform that allows selection of the most suitable one or more antibacterial compounds. Apart from using antibacterial components, generating cellular stress is also an achievable method for hydrogels to defeat infections.

Due to good biocompatibility and high porosity, hydrogels have been used to deliver antibiotics to overcome wound infections. Common antibiotics include fluoroquinolone, penicillin, cephalosporin, moxifloxacin, etc.^77^ Compared to oral delivery of antibiotics, a smaller dosage is needed for topical delivery via hydrogels to achieve an adequate antibiotic concentration in a wound site.^78,79^ Concerns about the usage of antibiotics are related to the fact that overdose or misuse of antibiotics may cause drug resistance. Additionally, antibiotics may also lead to normal mammalian cell death.^80^ Thus, to overcome drug resistance and cell toxicity, there is an urgent need to minimize the use of antibiotics.

**FIG. 3.**
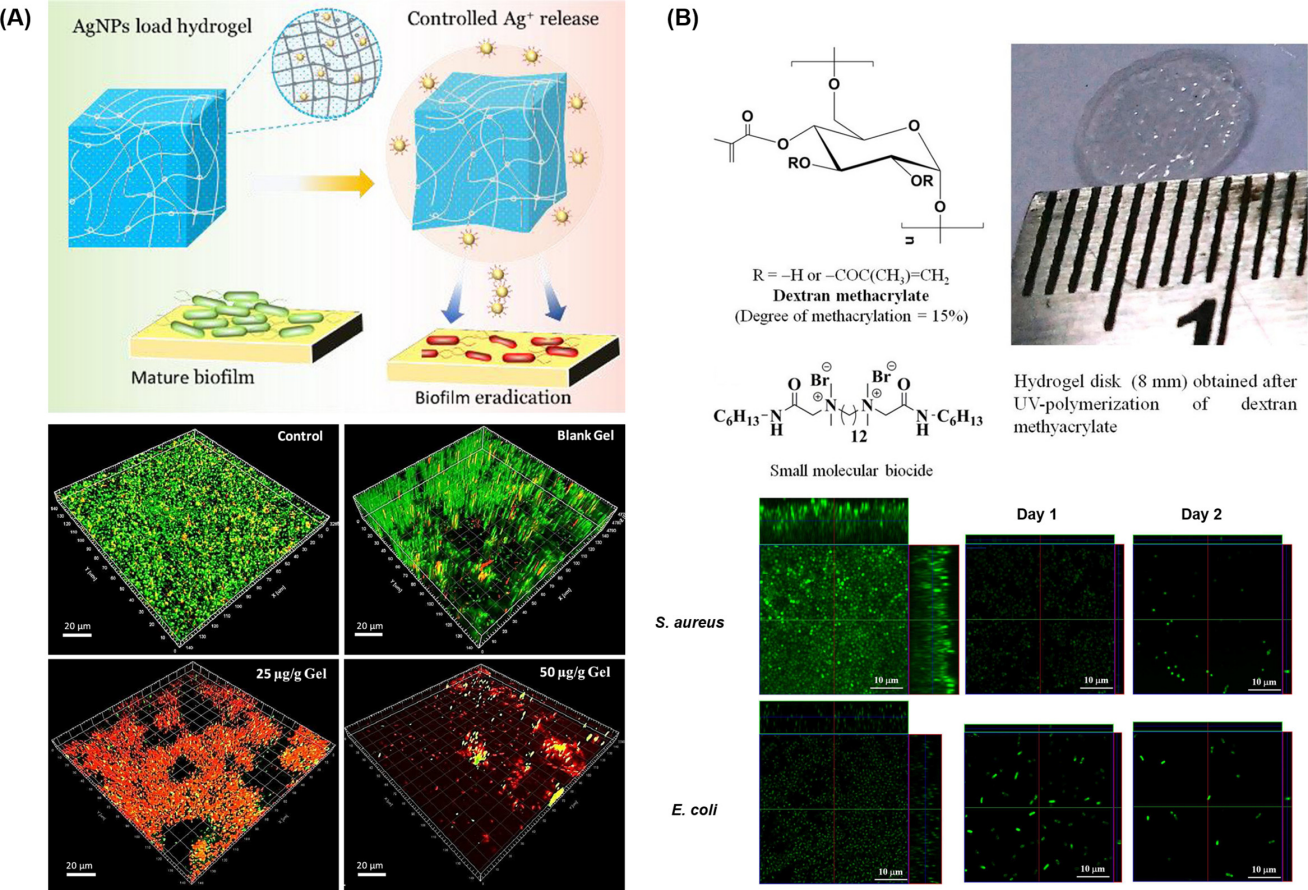
*In vitro* eradication of established biofilms using hydrogels loaded with antibacterial components: (a) different concentrations of ultrasmall silver nanoparticles (AgNPs) (25 and 50 *μ*g/g gel) were incorporated into Pluronic F-127 hydrogels to destroy the structures of *P. aeruginosa* biofilms.^84^ Reproduced with permission from Haidari *et al.*, ACS Appl. Mater. Interfaces **12**(37), 41011–41025 (2020). Copyright 2020 American Chemical Society. (b) Dextran methacrylate hydrogel was synthesized by photopolymerization polymer for encapsulating antibacterial cationic small molecules, which can penetrate the extracellular polymeric substances (EPS) of the established *S. aureus* and *E. coli* biofilms.^53^ Reproduced with permission from Hoque *et al.*, ACS Appl. Mater. Interfaces **9**(19), 15975–15985 (2017). Copyright 2017 American Chemical Society.

Many research approaches have used metal and metallic oxide nanoparticles as broad-spectrum antibacterial agents that may avoid antibiotic resistance, such as gold, silver, copper, magnesium, zinc oxide, and others.^44,81–83^ Metal ions can interact with bacterial cell membranes and subsequently inhibit bacterial DNA replication, leading to decreased bacterial viability.^44^ Haidari *et al.* homogenously dispersed ultrasmall silver nanoparticles (AgNPs, size < 3 nm) into a thermosensitive hydrogel, Pluronic F-127, to prevent AgNPs from fast oxidation.^84^ When AgNPs concentration reached 50 *μ*g/g in gels, 80% of the established biofilm was eradicated [Fig. 3(a)]. Notably, there was no apparent cytotoxicity toward human fibroblasts and human keratinocytes when co-cultured with their dressing exudates for 24 h.

Bacteriophages are a kind of human-safe virus found in nature that can halt bacterial infection.^85^ Therefore, loading hydrogels with phages is another promising strategy to treat infections for diabetic wounds with less concern of antibiotic resistance.^4^ The interaction process between bacteria and phages depends on the phage-type and bacterial host strain.^86^ For instance, phages λ, P1, and T4 can eliminate *E. coli.*^87^ Phages SAP-26 and KPO_1_K_2_ can remove *S. aure*us^88^ and *K. pneumoniae*,^89^ respectively. A single type of phage can be incorporated into hydrogel matrices.^90^ However, resistant bacterial variants may emerge as a side effect. Therefore, various strategies are employed, such as loading multiple phages in a cocktail or combining phages with antibiotics. For example, Kaur *et al.* fabricated polyvinyl alcohol (PVA) and sodium alginate (SA) based hydrogels for loading bacteriophages and antibiotics to treat resistant bacterial infections in wounds.^91^

Above-mentioned approaches are promising. However, challenges still exist. Metal ions and particles kill bacteria based on the release of leachables into the surrounding aqueous environment and thus may cause a tissue response.^92^ AgNPs were also witnessed to regenerate after dissolution into wound exudates with potential toxicity yet to be fully explored.^93^ The reportedly reduced bactericidal efficacy of AgNPs by serum albumin in human blood is another problem for metal ions and metal nanoparticles.^44^ The approach of loading bacteriophages in hydrogels to control biofilms in wounds is still in the early stage. Hydrogel dressing designs should take into account the release profile, penetrating ability, and bioactivity of bacteriophages. Similar to metal ions or nanoparticles and phages, cellular stress can also avoid drug resistance of traditional antibiotics.

Increasing numbers of antimicrobial-related research approaches use cellular stress to defeat bacteria. Like human cells, bacterial cells also suffer from cellular stress. The successful introduction of cellular stress can increase antibacterial efficacy. Abenojar *et al.* reported a biofilm disrupting hydrogel based on magnetic hyperthermia.^94^ When combined with amino acids, the system disrupts bacterial metabolism, demonstrating 85% disruption after 2 h treatment without causing toxicity to HeLa cells. In contrast, commercial antibiotics model drug vancomycin, when reaching its effective biofilm-disrupting concentration, showed extreme mammalian cell toxicity after 2 h treatment. Al-Bakri and Mahmoud embedded gold nanorods, utilizing its photothermal nature, into Pluronic F127.^95^ A 5.5–6.0 log reduction in bacterial viability was witnessed using near-infrared (NIR) laser irradiation. In addition to heat, ROS,^49,96^ light, ultrasound,^97^ and some mechanical damage^98^ can all be used to effectively kill bacteria in a broad spectrum without inducing drug resistance. It is worth noting that biofilms are a robust and dynamic system that is particularly challenging to eliminate. Bacteria living in the EPS matrix are commonly resistant to antibiotics. The drug resistance is mainly due to the diversity of the bacteria population and lack of physical contact during the host-immune response. Antibacterial factors can demonstrate universal bactericidal effects without physical contact and may better overcome the drug resistance when biofilms exist. All these nonspecific cellular stresses were proven to be broad-spectrum and do not rely on cell membrane recognition, nor are they thought to be susceptible to bacterial adaptation.

Nonspecific antibacterial effects often can work synergistically with EPS degrading/penetrating properties to break down a biofilm. When strong bactericidal effects are not paired with EPS degrading/penetrating properties, bacteria living inside EPSs may survive, leading to recurring infections. Moreover, to achieve satisfying antibiofilm effects, concentrations of bactericidal factors are usually much higher than their minimum inhibitory concentration (MIC). MIC of common commercial antibiotics is sometimes required to be as much as 16 to 512 times greater to show bactericidal effects against biofilms.^99^ When antibiotics reach a too high concentration, mammalian cell cytotoxicity becomes too strong to use clinically. Thus, potent and broad-spectrum antibacterial effects combined with EPS degrading/penetrating designs are preferable when developing an antibiofilm wound dressing.

To achieve EPS degrading properties, various research projects have targeted different aspects of biofilms. One strategy is designing EPS-penetrating hydrogels based on proximity in chemical structures. For example, amphiphilic small molecular antibacterial compounds have been investigated to disrupt negatively charged EPSs. Using dextran-based hydrogels encapsulated with a previously selected synthetic antibacterial cationic small molecule out of 18 related compounds from the same lab,^100^ 99.9% of established *S. aureus* and *E. coli* biofilms were eradicated [Fig. 3(b)].^53^ Another strategy is developing EPS-degrading hydrogels utilizing enzymes that can specifically target certain components in EPS. Proteolytic enzymes,^101^ oxidative enzymes,^102^ and polysaccharide degrading enzymes^103^ have been studied for this purpose.^104^ It should be noted that enzyme treatment has been widely used as an antibacterial strategy in the food industry.^105^ However, a combination of the antibacterial enzymes and hydrogels has not been fully demonstrated, especially the application of EPS degrading enzymes in wound dressings. Thallinger *et al.* loaded lysozyme in starch microgels and achieved a triggered-release microgel against alpha-amylase producing species.^104^ Lysozyme can degrade polysaccharide, which is not only the main component of bacterial cell walls, but also a building block of EPS matrix. Therefore, lysozyme would be predicted to have bactericidal and EPS-degrading properties. However, the lysozyme's action toward the EPSs was not investigated. To this end, the successful application of these EPS-degrading enzymes to break down, eradicate, and destroy biofilms in wound sites has great potential and is yet to be fully explored.

In summary, defeating existing biofilms is more challenging than preventing the formation of biofilms and requires more sophisticated designs. Killing the bacteria living within EPSs and breaking down the EPS matrix are both necessary to defeat existing biofilms. Potent and broad-spectrum bactericidal effects are essential due to the drug-resistant nature of the diverse bacteria population in biofilms. Thus, alternative antibacterial compounds and bacterial cellular stressors are preferred over antibiotics. Penetrating or breaking down already-formed EPS matrices is often combined with these antibacterial strategies to boost efficacy against biofilms. Hydrogels with similar chemical structures tend to penetrate biofilms better due to proximity in chemical structures. Enzymes are also effective strategies to loosen and break down EPS.

### C. Synergy between antifouling and antibacterial hydrogels

**FIG. 4.**
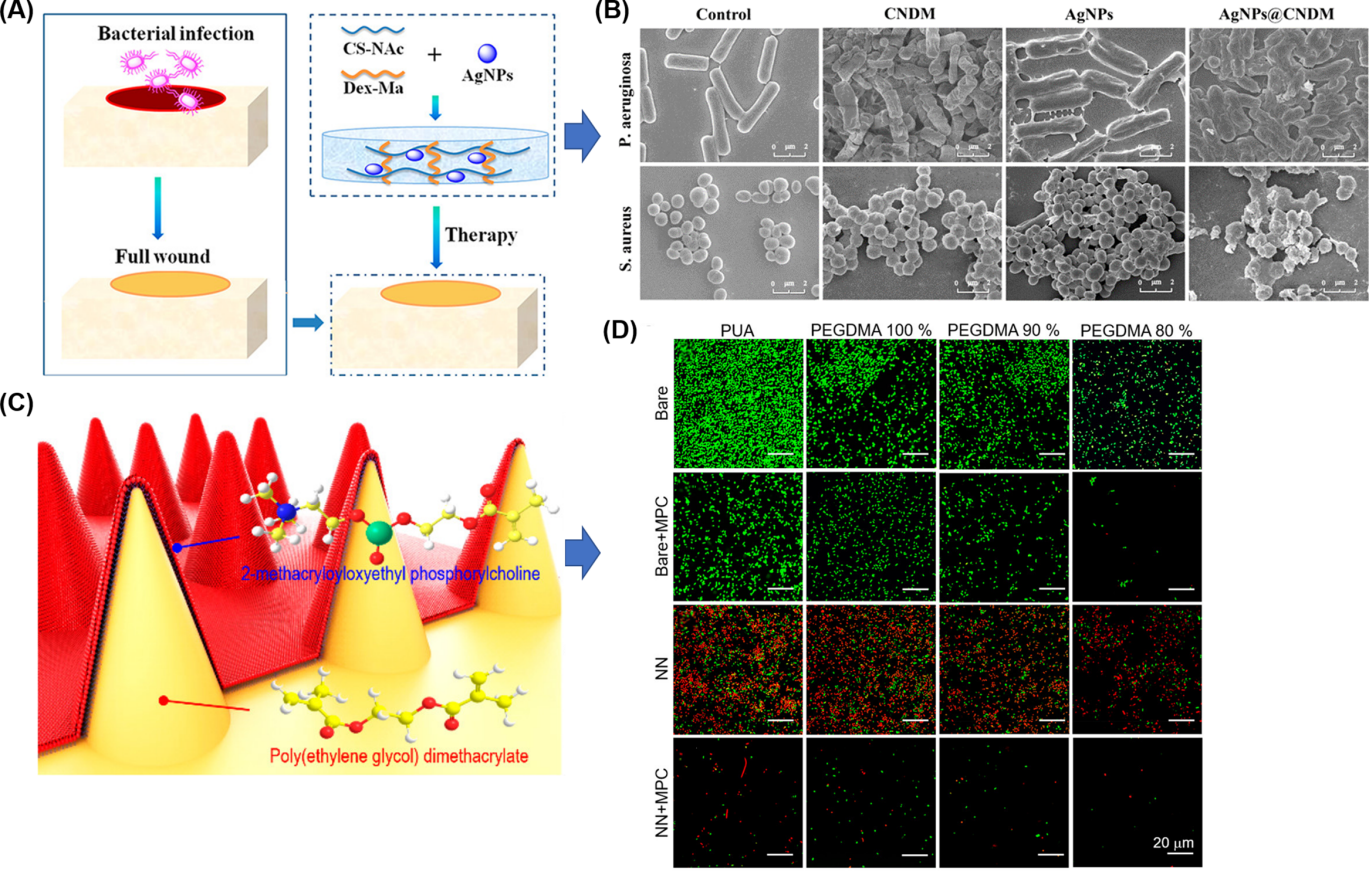
Synergy between antifouling and antibacterial hydrogels: (a) a zwitterionic hydrogel was obtained by simply adjusting the ratio between cationic chitosan and anionic dextran while incorporating AgNPs for enhancing their antibacterial activity, (b) the hydrogels with AgNPs showed superior antibacterial properties.^106^ Reproduced with permission from Shi *et al.*, Langmuir **35**(5), 1837–1845 (2019). Copyright 2018 American Chemical Society. (c) A hybrid hydrogel nanoneedle surface that can rupture cell membranes was fabricated based on zwitterionic polymers via a UV replica molding technique, providing antifouling and antibacterial properties, (d) *E. coli* was cultured on the 2-methacryloyloxyethyl phosphorylcholine (MPC)-grafted planar surface and MPC-grafted nanoneedle surfaces prepared with polyurethane acrylate (PUA) and different concentrations (80%, 90%, and 100%) of poly(ethylene glycol) dimethacrylate (PEGDMA). MPC-grafted nanoneedle surfaces prevented biofilm formation.^109^ Reproduced with permission from Park *et al.*, ACS Macro Lett. **8**(1), 64–69 (2019). Copyright 2018 American Chemical Society.

Bacterial attachment has been greatly reduced by using antifouling hydrogels, but elimination of initial bacterial attachment is rarely achievable only by antifouling hydrogels. Therefore, antifouling and antibacterial strategies are often combined and explored for the healing of diabetic wounds. For instance, Wu's lab developed a series of zwitterionic hydrogels with antifouling and antibacterial properties for wound dressing.^106^ The hydrogels were prepared via mixing thiolated chitosan and maleic acid-grafted dextran [Fig. 4(a)]. By adjusting the ratio between chitosan (positive charge) and dextran (negative charge) to achieve a net-zero charge of the composite, hydrogels with antifouling properties were achieved. After incorporating AgNPs, the antibacterial activity was enhanced and prolonged [Fig. 4(b)]. An antifouling strategy is not only helpful to prevent bacterial attachment, but also can prevent tissue adhesion to wound dressing.^107^ The bacteria were shriveled and damaged when the AgNPs were loaded into the gels. When applied *in vivo*, the combined dressing of hydrogel and AgNPs showed the lowest wound open area in Sprague Dawley (SD) male rats at both 7 and 10 days after treatment, significantly lower than when the same hydrogel without AgNPs or pure AgNPs were applied on their own.

Notably, the surface of hydrogels can also be designed into a nanopillar structure to kill bacteria via sharp tips rupturing the cell membranes.^108^ A combination of this nanotopography with antifouling polymers would create a surface with both antibacterial and antifouling properties. As Fig. 4(c) shows, the Jeong lab fabricated a nanoneedle surface of hydrogel based on poly (ethylene glycol) dimethacrylate (PEGDMA) and 2-methacryloyloxyethyl phosphorylcholine (MPC), which has an inherent antifouling nature.^109^ First, the PEGDMA hydrogel nanoneedle was prepared via a UV replica molding technique. After that, the PEGDMA needle surface was treated by O_2_ plasma and grafted with MPC to form a double layer nanoneedle surface. After 18 h of culture, colony-forming units (CFUs) of *E. coli* on the nanoneedle surface of MPC-grafted PEGDMA was reduced by more than two times compared to the planar surface of MPC-grafted PEGDMA [Fig. 4(d)], suggesting this nanotopography has superior antibacterial properties. It is noteworthy that the bactericidal effects of their material were achieved by surface modification rather than loading drugs. Their strategy is thus widely applicable to other hydrogels.

Moreover, to achieve the synergistic properties, hydrogels can be fabricated by compositing polymers with inherent antifouling or antibacterial properties. For example, Feng *et al.* fabricated a hydrogel based on ionic–covalent chitosan/poly (sulfobetaine methacrylate) (CS/PSBMA) with “Repel and Kill” properties, in which CS and PSBMA provided antibacterial and antifouling property, respectively. This hydrogel had lower than 8% of unspecific protein adsorption in phosphate-buffered saline (PBS) solution containing 1 *μ*g ml^−1^ horseradish peroxidase (HRP)-conjugated anti-IgG for 3 h. *In vitro* antibacterial test suggested that the small population of bacteria adhered to the hydrogel surface was killed by CS.^110^ Similarly, Peng *et al.* fabricated a CS/PEG dressing that prevented 98.8% of bacterial adhesion at an early stage and suppressed 93.3% of bacterial colonies for 7 days.^111^

### D. Smart hydrogels against infections

By using antifouling and antibacterial properties, smart hydrogels have been investigated to achieve the contact killing of bacteria attached to the surface and subsequent release of dead bacteria under proper stimuli. The release of dead bacteria is especially important for contact killing surfaces because dead bacteria can facilitate later attachment of bacteria. Furthermore, dead bacteria may also block antibacterial compounds from reaching live bacteria. By releasing dead bacteria when triggered, not only can the surface be self-cleaned, blocking of functional groups by accumulated foreign matter can also be avoided.^112^ Ionic strength,^113–115^ light,^58^ temperature,^116,117^ and pH stimuli^118^ can all be used to trigger release of the dead bacteria.

**FIG. 5.**
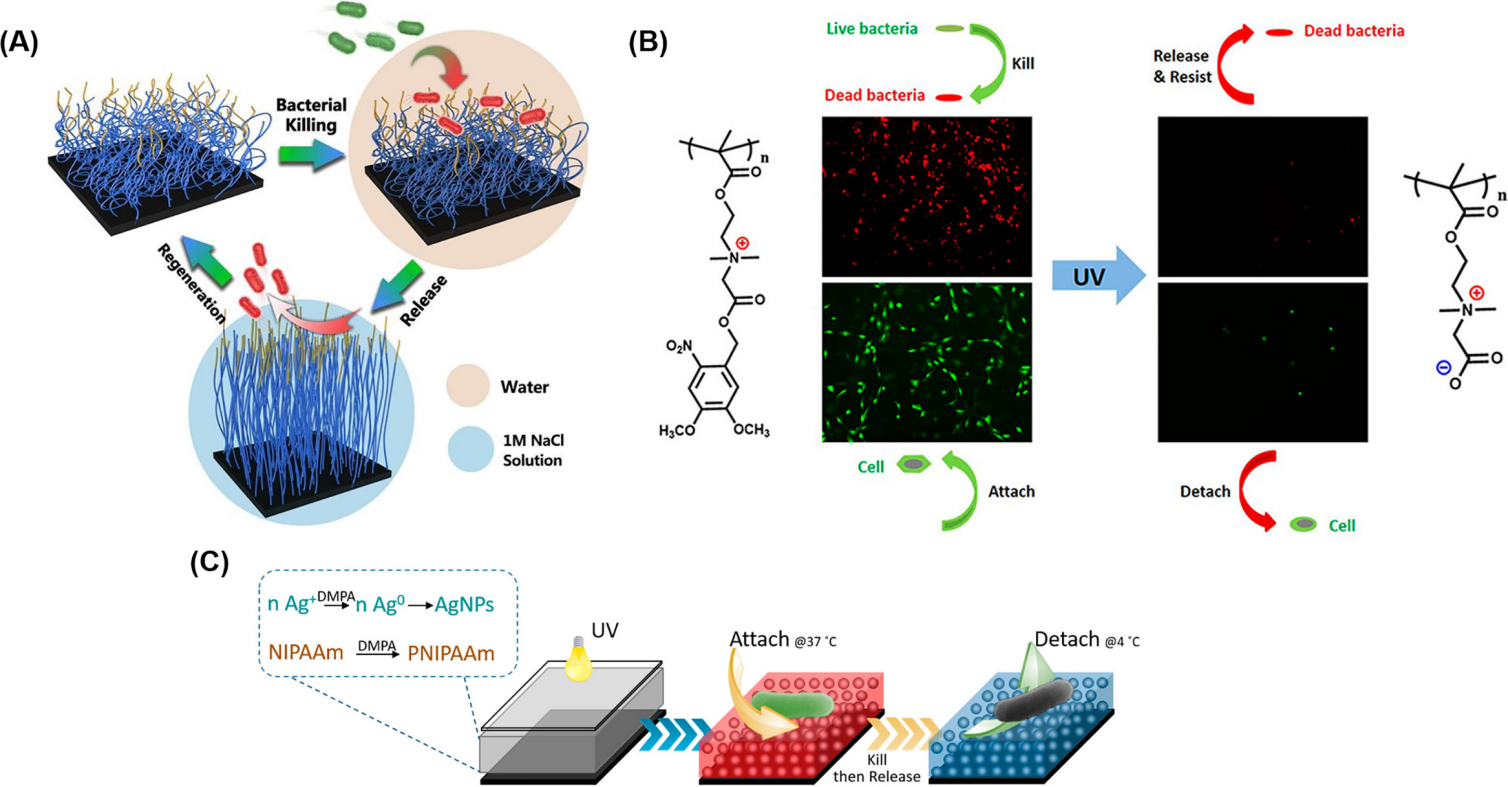
Smart hydrogels for antifouling: (a) using a salt-responsive surface based on a polyzwitterionic polymer, the contact angle decreased when the solution changed from water to 1 M NaCl solution; thus, bacteria were killed in water and released in salt water.^119^ Reprinted with permission from Huang *et al.*, Chem. Eng. J. **333**, 1–10 (2017). Copyright 2017Elsevier. (b) The surface of hydrogel based on photoresponsive polymer, poly[2-((4,5-dimethoxy-2-nitrobenzyl)oxy)-N-(2-(methacryloyloxy)ethyl)-N,N-dimethyl-2-oxoethan-1-aminium] (polyCBNA), was switched from cationic antibacterial to zwitterionic antifouling by UV treatment.^58^ Reproduced with permission from Liu *et al.*, Langmuir **35**(5), 1450–1457 (2019). Copyright 2018 American Chemical Society. (c) A thermosensitive poly(N-isopropylacrylamide) (PNIPAAm) hydrogel encapsulating AgNPs was prepared via a photopolymerization method. As temperature changed from 37 °C to 4 °C, the surface switched from killing to repelling bacteria mode.^117^ Reproduced with permission from Yang *et al.*, ACS Appl. Mater. Interfaces **8**(41), 28047–28054 (2016). Copyright 2016 American Chemical Society.

To achieve the smart functions for killing and desired release of bacteria, zwitterionic hydrogels can be designed for salt-triggered release of bacteria. The salt-triggered release of bacteria can be attributed to the conformational changes of polymers induced by ion-pairing interactions, electrostatic repulsion, and a fully wetting surface.^113^ The Yang Lab constructed a double-layered structure: poly[(trimethylamino) ethyl methacrylate chloride] (polyMETAC) and poly[2-(tert-butylamino) ethyl methacrylate] (polyTA) as the upper layer with bactericidal function was grafted on a salt-responsive zwitterionic polymer [poly(3-(dimethyl(4-vinylbenzyl) ammonio) propyl sulfonate), polyDVBAPS] bottom layer by covalent bond.^119^ The contact angle of polyDVBAPS decreased from more than 30° in water to approximately 10° in NaCl solution, enabling the switchable surface property to repel dead bacteria in salt solutions. In a typical process, bacteria could be absorbed and then killed in water, and finally removed in salt solutions [Fig. 5(a)]. The results showed that more than 93% bacteria killing efficiency was achieved. Importantly, ∼90% of attached bacteria could be released via 1.0 M NaCl solution, indicating the excellent regeneration capability for repeated usage. Alternatively, the triggered release of bacteria could be achieved using light. For example, Liu and Liu reported photo-responsive hydrogels containing a photolabile group 4,5-dimethoxy-2-nitrobenzyl and a quaternary ammonium group.^58^ The initial hydrogels killed bacteria due to the existence of quaternary ammonium compounds. After UV exposure, it switched to a zwitterionic antifouling state that repelled 97% of the dead bacteria [Fig. 5(b)].

To develop temperature-triggered release of bacteria, Poly(N-isopropylacrylamide) (PNIPAAm), a temperature-responsive polymer, has been extensively studied for controlling surface wettability and further inhibiting bacteria attachment. Its lower critical solution temperature (LCST) at 32 °C determines its phase change between hydrophilic and hydrophobic.^120^ As shown in Fig. 5(c), the Nie group fabricated a temperature responsive PNIPAAm hydrogel encapsulated with AgNPs via a one-step photopolymerization method. The existence of AgNPs kills bacteria attached to the hydrogels. When the temperature is switched from 37 °C to 4 °C, dead bacteria could be released from the surface of PNIPAAm. Because the temperature-responsive PNIPAAm undergoes a reversible phase change at its LCST transition, its hydrophilic adhesive interface switches to detach bacteria in a hydrophobic state.^117^ In addition, the Chen group developed a pH-sensitive surface based on a composite consisting of a pH-responsive polymer, poly (methacrylic acid) (PMAA), and a porous nanomaterial with lysozyme loaded as an antibacterial agent.^118^ In an acidic environment (pH = 4) for 0.5 h, the PMAA chains collapsed to facilitate lysozyme (10 *μ*g/ml) loading. When exposed to a physiological environment (pH = 7) for 2 h, PMAA chains were stretched, releasing the antibacterial agent and killing 95% of bacteria. In a basic environment (pH = 10) for 0.5 h, 90% of dead bacteria was repelled due to strong negative charges (COO^−^ group derived from COOH groups of PMAA) and high swelling of the PMAA chains. Similar to the salt-responsive surface, this pH-sensitive system can also be reused by reloading the antibacterial reagent.

The “kill and release” antifouling method had some achievements as discussed above. Some stimuli conditions, such as temperature or pH variation and salt concentration, seem to be gentle for the human body. However, their clinical application as antifouling wound dressings remains challenging because proper and controllable *in vivo* models to realize these stimuli is lacking. Additionally, the environment of diabetic wounds is complicated, including hyperglycemia, high oxidative stress, impaired angiogenesis, and high pH (7.0–9.0).^5,121,122^ In general, conditions in diabetic wounds often show changes at different stages of infections. Therefore, the “kill and release” hydrogel, triggered by multiexternal stimuli, might be needed and offer better control for the highly complicated *in vivo* environment.

## III. HYDROGELS AGAINST EXCESSIVE ROS

ROS plays several roles in both the normal and diabetic wound healing process.^123^ Signaling molecules of ROS include hydrogen peroxide (H_2_O_2_), hydroxyl radicals (^•^OH), superoxide anions (^•^O_2_^−^), nitric oxide (NO^•^), etc. Low ROS levels (H_2_O_2_ concentration in human plasma is below 10 *μ*M) can stimulate cell migration and promote angiogenesis.^124,125^ While ROS may have antibacterial activity, excessive ROS can hinder wound healing. Persistent ROS can prevent the transition from the inflammatory phase to the proliferation phase, subsequently aggravating wound infection.^19,126^ Unlike normal wounds, ROS can accumulate in diabetic wounds to a level that exceeds the cells' antioxidant capacity, leading to ECM degradation and cell death. It was reported that the concentration of H_2_O_2_ in normal human plasma ranged from 0.61to 6.79 *μ*M,^127^ with healthy cell metabolism producing an additional approximately 0.1 *μ*M of ROS. In contrast, the concentration of ROS can reach as high as 10–1000 *μ*M in a high oxidative stress environment.^18^ It was reported that H_2_O_2_ levels in type I diabetic plasma was 82.1 ± 31.4 *μ*M and that in type II diabetic plasma was 61.7 ± 39.1 *μ*M.^124,128,129^ Abnormally high amounts of ROS impair the functions of keratinocytes and dermal fibroblasts, leading to nonhealing wounds.

**FIG. 6.**
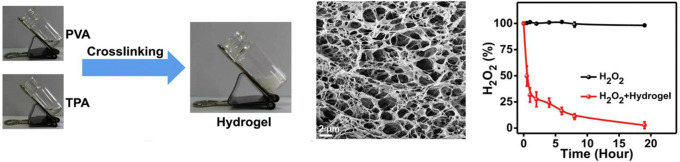
ROS-scavenging hydrogel: a polyvinyl alcohol (PVA) hydrogel was crosslinked by a ROS-responsive crosslinker (on the left). 100% of H_2_O_2_ was scavenged after 24 h, when the hydrogel was incubated in H_2_O_2_ (1 mM, 2 ml) solution (on the right).^131^ Reprinted with permission from Zhao *et al.*, Biomaterials **258**, 120286 (2020). Copyright 2020 Elsevier.

Therefore, antioxidant hydrogels could help eliminate excessive ROS in wound sites by scavenging free radicals, blocking free radical chain transfer, and relieving immune system dysfunction. Design of antioxidant hydrogels focuses on hydrogels as the vehicles of ROS scavengers, or hydrogels with inherent antioxidant properties, the latter of which could be achieved when synthesized using antioxidant macromolecules. Natural polyphenols, such as tannins, gallic acid, and curcumin, have been incorporated into many hydrogel systems to capture free radicals.^130^ Park *et al.* grafted gallic acid onto the backbone of gelatin (GGA) via horseradish peroxidase (HRP)-mediated reaction. GGA hydrogels scavenged more than 50% of 2,2-diphenyl-1-picrylhydrazyl (DPPH) radicals, higher than the pure gelatin hydrogel. In their mouse full-thickness skin defect model, the GGA hydrogel showed a higher wound closure rate than the pure gelatin hydrogel after 14 days.^123^ Moreover, the ROS content in the wound site can be used as a trigger for antioxidant release from hydrogels. Zhao *et al.* used a ROS-responsive crosslinker, N^1^-(4-boronobenzyl)-N^3^-(4-boronophenyl)-N^1^, N^1^, N^3^, N^3^-tetramethylpropane-1, 3-diaminium (TPA), to fabricate a polyvinyl alcohol (PVA) hydrogel that encapsulated functional drugs, including an antibiotic (mupirocin) and granulocyte-macrophage colony-stimulating factor (GM-CSF).^131^ 100% of H_2_O_2_ was scavenged after 24 h by the hydrogel when it was incubated with H_2_O_2_ (1 mM, 2 ml). When the hydrogel was degraded by H_2_O_2_, mupirocin and GM-CSF were released to treat the infected wound by decreasing excessive ROS and down-regulating pro-inflammatory cytokines (Fig. 6).

Compared to inherently antioxidant hydrogels, the hydrogels using ROS-responsive biomaterials are more attractive. The crosslinker can be degraded in response to ROS for scavenging it. In addition to scavenging ROS, the ROS-responsive biomaterials should have high biocompatibility and little inflammatory responses to the human body. In addition, its degradation rate should match with the change of ROS concentration in the surrounding environment. Thus, the structure and materials of the hydrogel should be deliberately controlled and chosen for the hydrogels to precisely respond to the dynamic ROS concentration. Moreover, ROS-scavenging treatment needs to be combined with antibacterial, angiogenic, or other functions for treating infected diabetic wounds.

## IV. HYDROGELS PROMOTING ANGIOGENESIS

Angiogenesis is a crucial step in forming new blood vasculatures, which supply oxygen and other nutrients to cells and tissues. However, unlike angiogenesis in normal wounds, the angiogenic process can be impaired by hyperglycemia, sustained inflammation, and ROS overproduction in diabetic wound sites. Once the regeneration of microvasculature is hindered, it can lead to nonhealing wounds.^132–134^ Thus, therapeutic angiogenesis is necessary. To promote angiogenesis, angiogenic growth factors, such as vascular endothelial growth factor (VEGF),^135,136^ can be delivered. Other promising approaches include the delivery of drugs or stem cells that upregulate genes related to angiogenesis, such as hypoxia-inducible factor-1-alpha (HIF-1α) and its downstream genes.^137^

However, growth factors are unstable in the diabetic wound environment, in which abundant proteases will degrade the native ECM and growth factors as well as their receptors.^5,138^ Therefore, the strategies to deliver growth factors play a crucial role in promoting diabetic wound healing. Hydrogels can be used as a vehicle to deliver exogenous growth factors into the wound bed. The hydrogel systems can protect the activities of growth factors allowing them to promote angiogenesis even in diabetic wounds. Fibroblast growth factor (FGF) is positively associated with the proliferation of endothelial cells and angiogenesis. For example, Hui *et al.* incorporated recombinant human acidic fibroblast growth factor (rh-aFGF) into a Carbomer 940 hydrogel to treat type II diabetic rats.^139^ The Carbomer 940 hydrogel was used as a carrier to maintain the efficacy of rh-aFGF during its delivery process. After 14 days, the healing rate of the group treated with Carbomer 940 hydrogel loaded with FGF reached 81.3%, which was higher than the control group (68.8%). Hematoxylin and Eosin (H&E) staining results suggested that more significant neovascularization was observed in the FGF-loaded group compared to the nonloaded control group.

**FIG. 7.**
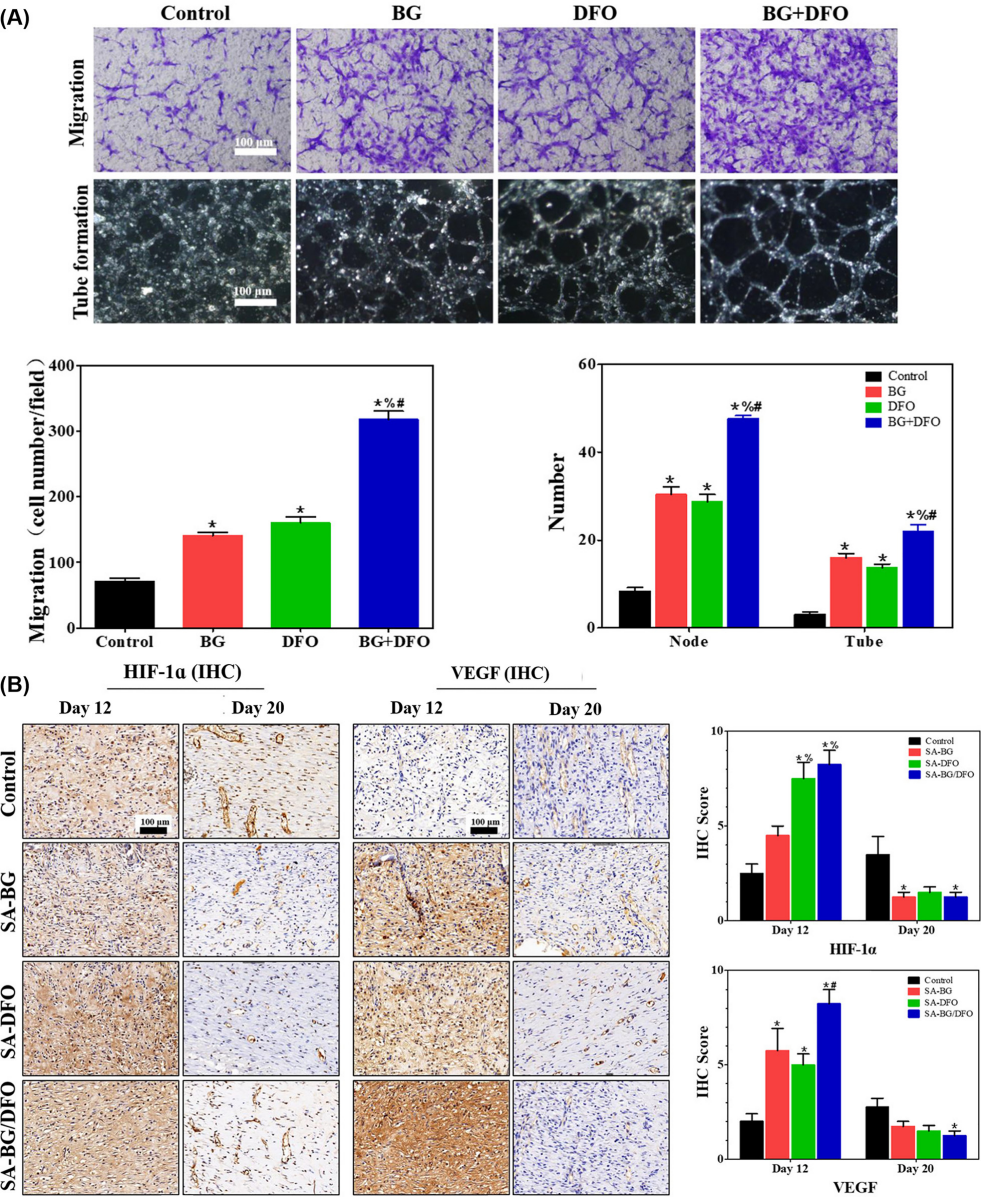
Hydrogel promoting angiogenesis: (a) the combination of desferrioxamine (DFO) and bioglass (BG) loaded sodium alginate (SA) hydrogels affected the migration and tube formation of HUVECs *in vitro* and (b) also promoted angiogenic factor expression *in vivo* (immunohistochemical staining for HIF-1α and VEGF).^142^ Reproduced with permission from Kong *et al.*, ACS Appl. Mater. Interfaces **10**(36), 30103–30114 (2018). Copyright 2018 American Chemical Society.

Furthermore, chemokines can also be loaded into the hydrogel for regulating angiogenesis.^134^ For instance, stromal-cell derived factor-1 (SDF-1) can attract endothelial progenitor cells (EPCs) to the wound bed to promote angiogenesis. The Ameer group developed a sustained release platform based on a temperature-sensitive Poly (polyethylene glycol citrate-co-N-isopropylacrylamide) PPCN hydrogel.^132^ SDF-1 was encapsulated into the system by gelation of PPCN solution at 37 °C. Sustained release of SDF-1 from the PPCN hydrogel to the wound site maintained a sufficient concentration and accelerated wound healing. The experimental model included full-thickness excisional wounds fixed by splints in diabetic mice. The wound closure rate of the SDF-1-loaded PPCN group reached about 60% at day 15. Optical coherence tomography (OCT) microangiography results suggested the blood vessel density of the SDF-1-loaded group was three times higher than that of the control group (treated with PBS). Moreover, some small molecule drugs can also be used to enhance angiogenesis. Desferrioxamine (DFO), an iron chelator, can bind free iron, mimicking oxygen deprivation in cells, thereby upregulating the expression of HIF-1α and its downstream gene VEGF.^134,140,141^ He *et al.* developed a sodium alginate (SA) hydrogel encapsulating DFO and bioglass (BG), which promoted human umbilical vein endothelial cell (HUVEC) migration and tube formation after 4 h [Fig. 7(a)]. A diabetic rat model was established to evaluate the efficacy of the hydrogel. Immunohistochemical (IHC) stained images of SA-BG/DFO group showed the highest expression of HIF-1α and VEGF, which were directly correlated with neovascularization [Fig. 7(b)].^142^

Mesenchymal stem cells (MSCs) can secrete many growth factors, i.e., bFGF and TGF-β1, that are beneficial for chronic wound healing. However, their survival rate is often low, sometimes less than <10% 24 h after being directly injected into the wound bed.^143,144^ Researchers encapsulated MSCs into hydrogels to improve their survival rate after transplantation.^145^ Zhang *et al.* fabricated an MSCs-loaded thermal sensitive hydrogel based on PNIPAAm to treat diabetic ulcers. The cell-laden hydrogel promoted angiogenesis, granulation tissue formation, re-epithelialization, and even the regeneration of hair follicles and sebaceous glands for diabetic wound healing.^146^ The average unhealed area of type II diabetic mice was 24.6% ± 4.21% for the hydrogel loaded MSCs group at day 7, which was much lower than that of the untreated control group (79.54% ± 5.92%). Masson trichrome staining results confirmed that MSCs led to the formation of more blood vessels. Notably, adipose-derived stem cells (ASCs) are easy to isolate from adipose tissue and have been applied in many clinical trials for diabetic wound healing.^147^ ASCs can secrete cytokines that have immunomodulatory effects.^143,148–151^ Gurtner lab developed an injectable poly (ethylene glycol) PEG–gelatin hydrogel as a vehicle to deliver ASCs and treat diabetic ulcers. The ASC laden hydrogels enhanced angiogenesis of the wound and accelerated wound closure.^144^ Moreover, many other cell types, such as endothelial cells and bone marrow mononuclear cells, can also be loaded into hydrogel matrices for diabetic wound healing.^152–154^

As discussed above, the hydrogel platform for promoting angiogenesis and wound healing achieved some effectiveness in animal models. Despite this success, few products have been tested in clinical trials. Ultimately, it is difficult to treat diabetic wounds using a hydrogel with only a single function. In the following section, multifunctional hydrogel dressings will be discussed with a particular focus on their antibacterial performances and *in vivo* healing results.

## V. MULTIFUNCTIONAL HYDROGELS DRESSINGS

As indicated in previous sections, due to the multifactor nature of diabetic wounds, single-function hydrogels usually fail to support complete healing. Based on this, researchers developed multifunctional dressings to achieve better wound healing outcomes by addressing multiple needs. Among the above-mentioned strategies, anti-infection is the most investigated, because infections not only widely appear in diabetic wounds, but also largely hinder the healing process. Therefore, most multifunctional dressings have control of infections as one aspect. Additionally, glucose control is a commonly used strategy due to the high glucose environment of diabetic wounds. Angiogenesis, as a key factor during wound healing assessment, is also often combined with antibacterial functionality in hydrogel dressing designs. Finally, on-demand wound dressings are being increasingly investigated as a new approach to improve multifunctional wound dressings.

When evaluating a multifunctional dressing, antimicrobial effectiveness is a key parameter. Angiogenesis and epithelialization are the following two most evaluated parameters to quantify wound healing besides observational wound bed closure. In addition, collagen deposition and wound healing-related gene expression are also used to evaluate wound healing. In this section, multifunctional hydrogel dressings are discussed, evaluated, and compared mainly based on antimicrobial performances, angiogenesis and epithelialization, and other featured performances, with a particular focus on anti-infection effects and *in vivo* healing results.

### A. Glucose control and anti-infection

High blood glucose is a key cause of infected diabetic wounds. In the clinic, administration of insulin or other drugs is a common strategy of wound management. However, when wounds develop into chronic wounds, deficient local vasculature makes it harder to deliver intravenous or systemic treatments. High glucose concentration in wound sites can assist bacterial growth and development, inhibiting the wound healing process.^155^ Thus, local administration of insulin or other glucose-control reagents is a potentially impactful strategy.

**FIG. 8.**
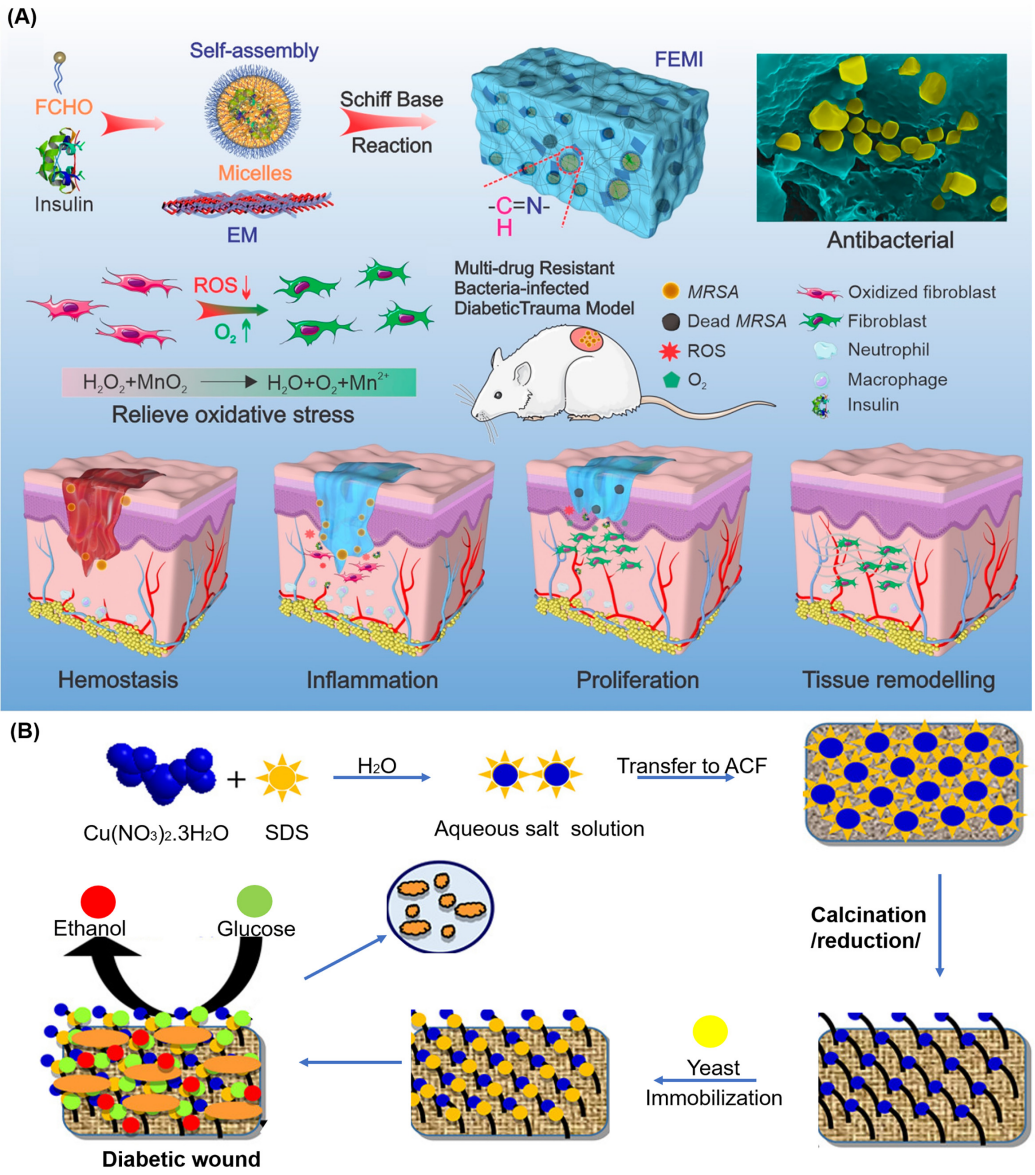
Multifunctional hydrogels for diabetic wounds with persistent infections: (a) a schematic diagram illustrating the injectable, self-healing, and adhesive hydrogel (FEMI) hydrogel for multidrug-resistant (MDR) bacteria-infected diabetic wound healing. After loading insulin and MnO_2_ nanosheets, the hydrogel can regulate blood glucose and relieve oxidative stress of wounds.^30^ Reproduced with permission from Wang *et al.*, Nano Lett. **20**(7), 5149–5158 (2020). Copyright 2020 American Chemical Society. (b) A preparation scheme of a yeast immobilized and copper nanoparticle incorporated wound dressing, the dressing can produce ethanol by consuming glucose, helping to control bacteria growth.^156^ Reproduced with permission from Bhadauriya *et al.*, ACS Appl. Bio Mater. **1**(2), 246–258 (2018). Copyright 2018 American Chemical Society.

Wang *et al.* demonstrated injectable multifunctional hydrogels [Fig. 8(a)].^30^ Superior antibacterial capability against multidrug-resistant (MDR) bacteria was achieved by combining the synergistic effects of antimicrobial peptide ε-polylysine and MnO_2_ nanosheets. Meanwhile, the pH/redox dual-responsive hydrogel Pluronic F127 can regulate blood glucose by sustained release of insulin. The so-called injectable multifunctional (FEMI) hydrogel caused an almost 100% decrease in bacterial colonies for *E. Coli*, *S. aureus* and methicillin-resistant *S. aureus* (MRSA). The mechanism is based on electrostatic attraction between the positive charged hydrogel and negatively charged bacterial cell membranes, working synergistically with the nano knife-like MnO_2_ nanosheets to disrupt bacterial cell membranes. Mechanisms such as these do not involve small molecule antibiotics and are thus less likely to encounter or induce drug resistance. When applied *in vivo*, the FEMI hydrogel led to 78.2% wound closure on day 7 in diabetic mice, which was higher than the PBS control group (39.9%), small molecule antibiotics treated group (44.1%), and the unloaded blank hydrogel group (58.1%).

Similar to Wang *et al.*, other researchers focused on topical insulin delivery to regulate wound site glucose. However, insulin may experience loss of functionality if simply added to a hydrogel system. A more robust and reliable system was therefore desired. Bhadauriya *et al.* designed a nanofiber system that can control glucose and infections simultaneously [Fig. 8(b)].^156^ Unlike topical insulin delivery, where insulin stability may be maintained by nanoparticles,^30^ a yeast extract can be immobilized onto a surface. The copper–carbon nanofiber system with surface-immobilized yeast extract effectively consumed up to 44% of free glucose. In addition, it showed no significant influence on overall blood glucose when tested *in vivo*, ensuring the safety of the wound dressing system (i.e., mitigating the risk of acute hypoglycemia). Both gram-positive and gram-negative bacteria showed complete inhibition when treated with copper–carbon nanofiber dressing. Compared to commercial insulin, which usually requires 2–8 °C for storage, yeast extract can stay active for up to 4 years under ambient temperature.^157^ The idea of designing a noninsulin glucose regulating system makes dressings easier to store than systems loaded with insulin.

### B. Promoting angiogenesis and anti-infection

**FIG. 9.**
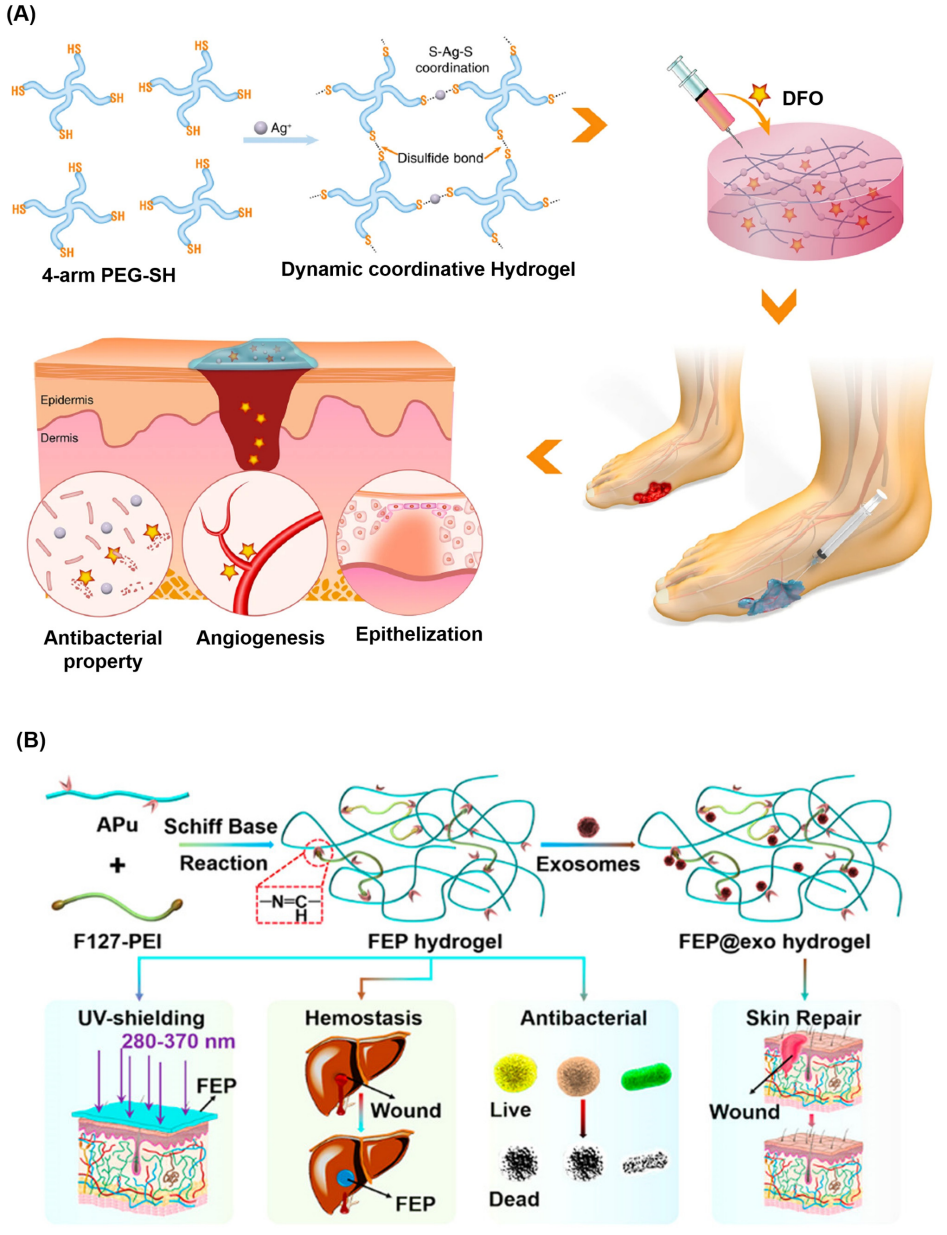
Hydrogels for promoting angiogenesis and anti-infection: (a) self-healing Ag(I)-thiol (Au–S) coordinative hydrogel was developed by mixing four-arm-PEG-SH with AgNO_3_ that possessed antibacterial, pro-angiogenesis, and pro-epithelization properties.^161^ Reproduced with permission from Chen *et al.*, NPG Asia Mater. **11**, 3 (2019). Copyright 2019 Authors, licensed under a Creative Commons Attribution (CC BY) license. (b) Schematic illustration of multifunctional polysaccharide-based (FEP) hydrogel loaded with exosomes to accelerate diabetic wound healing.^162^ Reproduced with permission from Wang *et al.*, ACS Nano **13**(9), 10279–10293 (2019). Copyright 2019 American Chemical Society.

As mentioned in Sec. IV, facilitating angiogenesis and revascularization is also a common strategy to promote wound healing and is often combined with bactericidal components to treat diabetic wounds.^158–160^ Chen *et al.* reported an injectable hydrogel developed by mixing four-arm-PEG-SH with AgNO_3_ nanoparticles as shown in Fig. 9(a).^161^ The dressing displayed an antibacterial nature against *S. Aureus*. Upon *in situ* encapsulation of desferrioxamine (DFO), an angiogenic drug, a decrease in wound area by 50% of original size at day 7 was achieved in SD rats, higher than both the untreated control group and pure hydrogel group, which decreased by 30% at day 7. The Lei lab reported an angiogenic-promoting hydrogel dressing with antibacterial properties [Fig. 9(b)].^162^ They took advantage of the angiogenesis-promoting effect of mesenchymal stem cell-derived exosomes and the antibacterial nature of pullulan to form an injectable, pH-responsive, self-healing, and sticky hydrogel. Pluronic F127 grafted polyethyleneimine (PEI) and aldehyde pullulan was crosslinked by the Schiff base reaction to form the so-called injectable adhesive thermosensitive multifunctional polysaccharide-based (FEP) hydrogel. The FEP hydrogel achieved a bactericidal effect against gram-positive, gram-negative, and drug-resistant strains, all of which were more profound than ampicillin, a small molecule antibiotic. Their FEP hydrogel, when combined with exosomes, achieved a wound closure rate around 60%, not only higher than the untreated control, but also higher than either the hydrogel or exosome used individually. Moreover, immunofluorescence staining showed about two times more blood vessels formed in wound tissues treated with the FEP-exosome hydrogel than that formed in wound tissues treated with only exosomes, indicating good compatibility of exosomes with the hydrogel system. Interestingly, the use of stem cell-derived exosomes achieved an outstanding result despite the existence of high blood glucose. The wound healing was improved in various aspects including re-vascularization, collagen deposition, re-epithelialization, less scar formation, and better appearance of skin appendages.

### C. On-demand wound dressings

**FIG. 10.**
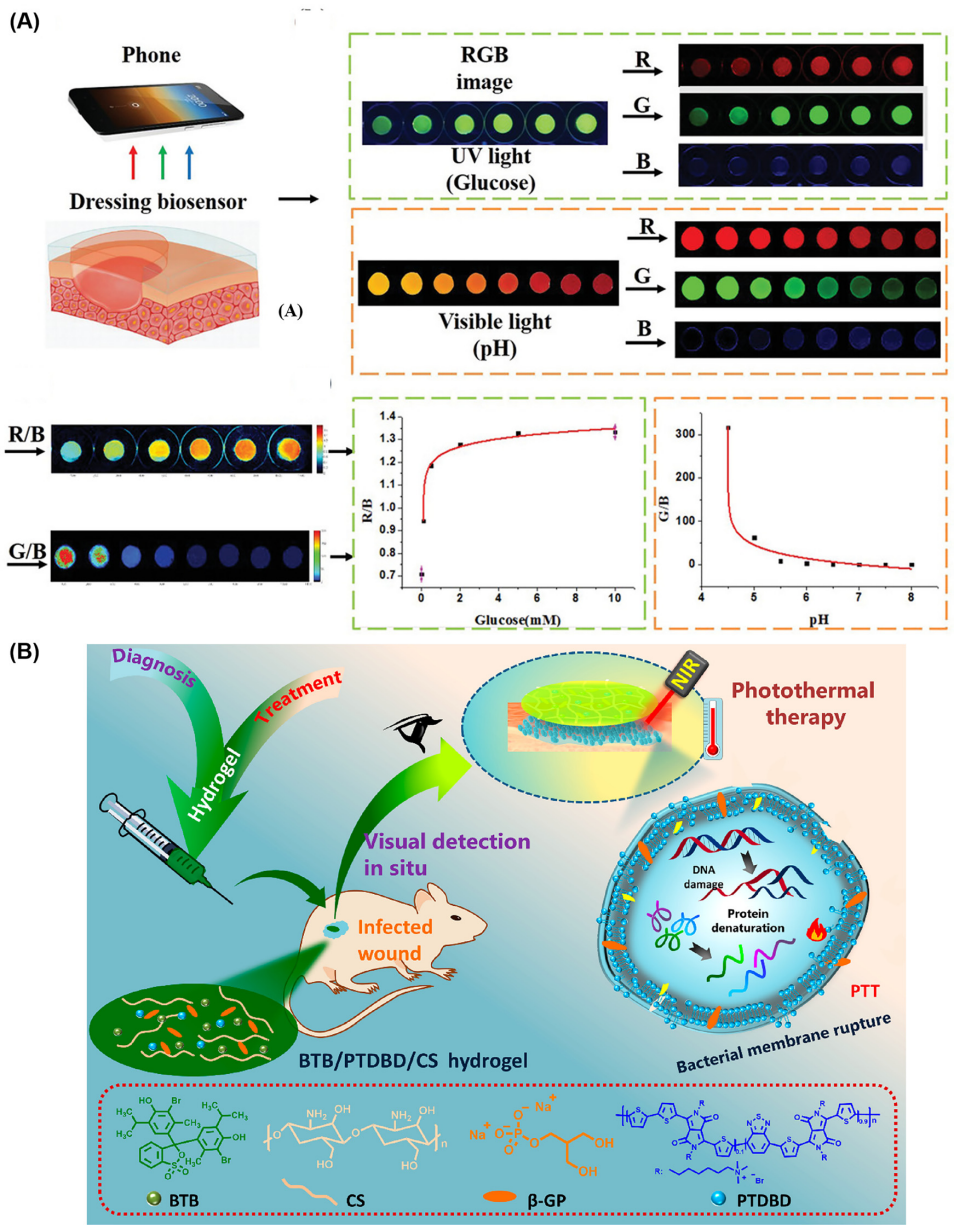
Smart hydrogel wound dressings that sense, monitor, and interact with wounds while treating chronic infections in diabetic wounds: (a) glucose-sensing enzymes that can react in proportion to glucose concentration and a pH dye were immobilized into an anti‐biofouling zwitterionic hydrogel, which provided a moist healing environment for diabetic wounds. Meanwhile, glucose concentration between 0 and 10 mM and pH value (4.0–8.0) in wound microenvironment can be monitored.^121^ Reproduced with permission from Zhu *et al.*, Adv. Funct. Mater. **30**(6), 1905493 (2020). Copyright 2019 Wiley‐VCH Verlag GmbH & Co. KGaA, Weinheim. (b) A thermosensitive chitosan (CS)-based hydrogel encapsulating pH-sensitive dye and near-infrared (NIR)-absorbing conjugated polymer can detect the pH change of an infected wound. Then photothermal therapy after *in situ* visual diagnosis.^166^ Reproduced with permission from Wang *et al.*, ACS Appl. Mater. Interfaces **12**(35), 39685–39694 (2020). Copyright 2020 American Chemical Society.

In wound dressings, some functionalities may be required at different levels throughout the wound healing progress. For example, antibacterial effects are only needed when an infection exists, and in the ideal case, the dose would adapt to current bacterial loads. Similarly, glucose control mechanisms should scale with local glucose concentrations. Some systems can give visual indications of certain relevant variables in wounds. Compared to current diagnostic processes, patients and care providers desire to know the status of the wounds easier, cheaper, and quicker: are the wounds infected? If so, how intense is the infection? Therefore, smart sensing wound dressings that can deliver functions on-demand are desirable, especially for chronic wounds, such as diabetic wounds. While focusing on managing wounds from multiple angles, researchers recently started to explore the next-generation wound dressings that can sense, monitor, and interact with wounds. Specifically, the glucose level is the most widely monitored factor in diabetic wound dressings. Zhu *et al.* successfully monitored the glucose level and wound healing process while providing wound healing and antifouling functionalities.^121^ The zwitterionic poly-carboxybetaine (PCB) hydrogel matrix has an antifouling nature that can largely prevent bacteria from attaching to the surface. The dressing can sense glucose across a range of 0.1–10 mM. Due to the immobilization of glucose-sensing enzymes (glucose oxidase and horseradish peroxidase), the fluorescence intensity of the hydrogel can be detected by UV light under 365 nm, which is positively correlated with the glucose level. A pH indicator dye (phenol red) was also incorporated into the PCB hydrogel for observing the pH changes in a range of 4.0–8.0 during diabetic wound healing process [Fig. 10(a)]. Thet *et al.* developed an intelligent hydrogel dressing showing strong fluorescence in the presence of a biofilm.^163^ Qiao *et al.* monitored infection taking advantage of a pH-responsive fluorescence resonance energy transfer (FRET) transition between Cyanine3 and Cyanine5 and treated by NIR-triggered release of antibiotics.^164^ More recently, researchers also improved the sensor properties to gain visual color change so that the diagnostics can be even faster, cheaper, easier, and require less equipment. Along these lines, Zepon *et al.* used a mix of natural compounds: carrageenan, locust bean gum, and cranberry extract, to monitor wound status by showing visible color change.^165^

**FIG. 11.**
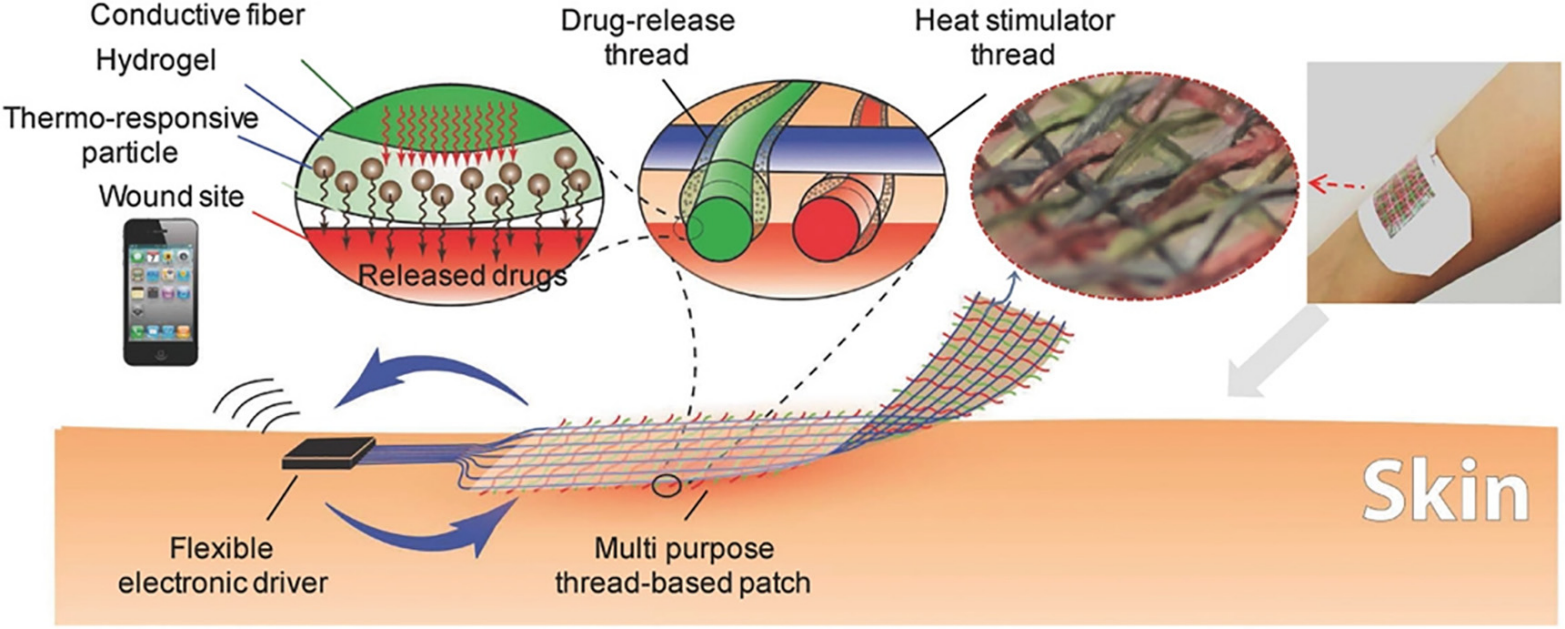
On-demand wound dressing: electrically driven responsive thread-based antibacterial wound patch with conductive, thermo-responsive, and drug-releasing properties.^167^ Reproduced with permission from Mostafalu *et al.*, Adv. Funct. Mater. **27**(41), 1702399 (2017). Copyright 2017 Wiley‐VCH Verlag GmbH & Co. KGaA, Weinheim.

All the above-mentioned research projects either did not or paid little attention to reacting to the signals the dressings sensed, the most important purpose of detecting them. Nevertheless, some researchers have successfully combined the smart dressing idea with effective antibacterial strategies. Feng's group developed a theragnostic hydrogel combining *in situ* visual diagnosis with NIR triggered photothermal therapy [Fig. 10(b)].^166^ By incorporating a pH-sensitive bromothymol blue (BTB), the hydrogel showed visible color changes corresponding to various pH levels in the infected wound microenvironment. Based on a naked-eye diagnosis, photothermal therapy triggered by NIR light can be applied if needed. They reported a less than 10% bacterial viability within 8 min upon laser irradiation. When applied *in vivo*, the elevated temperature caused by photothermal therapy did not result in disturbed structures. Mostafalu *et al.* developed a textile dressing composed of separate drug-release and heat stimulator threads (Fig. 11).^167^ Each of the cotton threads was coated with a conductive carbon ink as a core microheater and an alginate/poly (ethylene glycol) diacrylate hydrogel loaded with functional components. An Arduino microcontroller can be used to drive an electrical voltage (1.5–5 V) that was applied on the microheater to generate heat (temperature range 35–45 °C). Interestingly, a smartphone can wirelessly transfer commands to the microcontroller connecting this on-demand drug delivery platform. Thermo-responsive particles prepared by poly(N-isopropylacrylamide) (NIPAM) with a critical temperature of ∼40 °C were encapsulated into the hydrogel components and used to achieve the desired release of drugs. Notably, this system could possibly be integrated with a glucose sensing system to achieve sensing and thus on-demand delivery of desired drugs. In addition, the system is highly tunable to load and deliver antibiotics and pro-healing factors. When tested *in vitro*, cefazolin led to an approximately 10^7^ of reduction in colony-forming units (CFU) for *E. Coli* and *S. Aureus*. The VEGF-loaded patch showed more than a threefold increase in granulation tissue deposition compared to controls after 10 days of treatment in a diabetic mouse model.

Overall, multifunctional hydrogel dressings are designed to achieve improvements in multiple aspects of infected diabetic wounds. Many of the strategies reviewed here were able to demonstrate design effectiveness either *in vitro* or *in vivo*. Despite the fact that most of the targeted aspects of wound healing were improved, the overall outcomes were only minimally improved. The lack of satisfying synergistic results could be due to unknown interactions in the wound healing process that are made more complicated by the diabetic condition. A misalignment in time may also be a key reason: different stages of wound healing have different requirements. Instead of a tuned sequential delivery, most of the multifunctional hydrogel dressings discussed here delivered different components all at once. Despite the less satisfying synergistic results on wound healing, the responsiveness of on-demand hydrogel dressings achieved satisfying performances. Monitoring features meet the needs of both patients and care providers by adding quick diagnosis functionality to the dressings. Because diabetic wounds often require close monitoring and timely treatment, such real-time diagnosis could be a first alert that a wound management strategy requires adjustment.

## VI. OUTLOOK

In general, a successful all-in-one wound treatment hydrogel that combats biofilms while ameliorating other effects of diabetes is yet to be developed. Certain single-function hydrogel dressings, including those that are antifouling, antibacterial, and pro-healing, can all achieve a relatively satisfying result in the aspect of focus. However, once applied *in vivo*, it usually has been difficult to achieve good overall wound closure results compared to normal wound healing process. This is mainly because single-function hydrogel dressings failed to improve an overall wound condition due to multiple interconnected causes of nonhealing wounds. Even hydrogel dressings that claim to have multiple functions cannot always achieve equal effectiveness in every aspect. For example, pro-healing and antibacterial dual-functional dressings are often relatively ineffective on healing outcomes. The lack of full effectiveness sometimes results in little improvement in ultimate wound closure performance. A lack of close contact is another general shortcoming of many kinds of dressings when used as a treatment by itself. Any unreachable area will become a “blind spot” of treatments. *In situ* gelation and injectable hydrogels can improve the reachability of hydrogels since they can adapt to the wound topography to contact the blind spots. Currently, the most common applications of hydrogels in diabetic wounds are as a drug delivery system and a short-term dressing. High drug loading efficiency and controllable release for hydrogel treatments still need to be improved. In addition, suitable mechanical strength and excellent biocompatibility are all important requirements to treat diabetic wounds that have abnormal inflammation, poor angiogenesis and low levels of growth factors.

A typical clinical treatment process of diabetic wounds includes removal of chronic hyper-granulation tissue, wound debridement, off-loading, surgery, and finally using a wound dressing.^14,15^ From a clinical perspective, once a biofilm forms, the treatment becomes challenging.^168^ Thus, it may be more promising to prevent biofilm formation using an antifouling hydrogel rather than removing an established biofilm. In addition, it takes a long time to heal or treat infected diabetic wounds using antibiotics due to the drug resistance of bacteria. To avoid drug resistance, minimizing the dosage of antibiotics or other antibacterial agents through developing hydrogels as a delivery platform may be more practical than exploring new antibiotics. Moreover, FDA-approved biomaterials as hydrogel wound dressings are limited for the treatment of diabetic ulcers. Successful application of the hydrogels to clinical treatment of diabetic ulcers still remains challenging with many studies still in the early stage. Some examples of the hydrogels in clinical trials for diabetic ulcer healing are listed in Table I.

**TABLE I.** Clinic trial status of hydrogels for diabetic wounds.^169^

Hydrogels	The stage of a clinical trial	ClinicalTrials.gov identifier
Honey-based hydrogel	Phase 1	NCT03816618
Carbopol-based hydrogel containing erythropoietin	Phase 2	NCT02361931
Placebo hydrogel containing plant extracts	Phase 2	NCT01427569
Doxycycline monohydrate hydrogel	Phase 2	NCT00764361
Acellular porcine dermal matrix	Phase 3	NCT01353495
Human allograft amniotic membrane	Phase 4	NCT02120755
Woulgan gel	Phase 4	NCT02631512

As there are still unmet needs in clinical diabetic wound management, the easy-to-monitor smart dressing brings a new trend into hydrogel dressing development. A diagnostic dressing that indicates a change of wound status makes diagnosis faster, easier, and cheaper. Personalized treatments of infections also become possible based on smart wound dressing technologies. Because patients often fail to realize the severity of their wounds, close monitoring and timely treatments may be able to prevent unhealing wounds from developing. However, the wound status detection signal is not sensitive enough for certain dressings to respond promptly, advanced wound monitoring is highly desirable in next-generation hydrogel wound dressings.

With the increasing need for chronic wound management driven in part by a large population of diabetic patients, developing anti-infection hydrogels with multiple functions holds excellent potential for accelerating diabetic wound healing and improving patients' quality of life.

## AUTHORS' CONTRIBUTIONS

D.G. and Y.Z. contributed equally to this work.

## Data Availability

Data sharing is not applicable to this article as no new data were created or analyzed in this study.
